# Death of the Lower Extremity from a Tight Bandage

**Published:** 1858-09

**Authors:** R. H. Dalton

**Affiliations:** Aberdeen Mississippi


### BUFFALO MEDICAL JOURNAL

AND

### MONTHLY REVIEW.

**VOL. 14.** SEPTEMBER, 1858. **NO. 4.**

### ORIGINAL COMMUNICATIONS.

ART. I.—*Death of the Lower Extremity from a Tight Bandage.* Letter from Dr. DALTON, of Mississippi, addressed to Dr. HAMILTON.

ABERDEEN, Miss., Sept. 23d, 1856.

F. H. HAMILTON, M. D.,

Dear Sir: Your letter of the 9th inst., requesting me to favor you with all the facts in the case of a negro slave of Mr. A. B., of S. C., Ala., whose thigh I amputated after fracture in March, 1838, has been kindly forwarded to me by the postmaster at L., where I resided at the time. I regret that I have no record of the case at this time, and must, therefore, rely on memory alone for such facts as I shall be able to state.

The boy was about 16 years old, well grown and of good constitution. His thigh had been fractured about three or four inches below the great trochanter, say fourteen or fifteen days before I was called to see him. A young physician, a graduate of Transylvania University, was called in and applied the *bandage* as taught at that time by Dr. Dudley. My impression is that no splints were used, and that the bandage reached from the toes to the groin, until a few days before I saw him, when that part embracing the upper part of the thigh was removed. I do not recollect whether the bandage was on when I arrived; but the whole extremity was found in a state of dry gangrene with the exception of the upper two-thirds of the thigh, which was enormously swollen and partially gangrenous as high up as the groin. The negro was greatly emaciated; countenance stupid and dejected; pulse exceedingly feeble; and he seemed to be totally indifferent as to his fate. After a careful examination of the case, I stated, as my opinion, that nothing could be done; that if amputation were attempted, the patient would die during its progress; and in that opinion I was sustained by the late Dr. Robert L. Hunter, of L., Ala., who then resided in a neighboring town, and was present to assist me. The owner, however, insisting that the limb should be amputated, even at the hazard of death, and assuming the whole responsibility of the result, the boy was placed upon a table and the operation commenced with a scalpel, Dr. Hunter compressing the femoral artery on the ramus of the pubis by his thumb, there being no room for a tourniquet. The section was made as near as possible to the ramus of the pubis, inasmuch as the gangrene seemed to occupy the whole thigh. The cutting was done as boldly and rapidly as practicable, for the purpose of avoiding exhaustion; but much time was consumed in securing the numerous arteries which were necessarily divided in passing through such a bulk of muscle, occupied as they were by large recurrent vessels. Great care was taken to avoid hæmorrhage, but when the femoral artery was divided, a gush of blood was nearly fatal to the patient. It was instantly arrested, however, by additional pressure, while the ligature was quickly applied. At this stage of the operation, the pulse at the wrist ceased, and some moments were lost in administering brandy to the patient. Resuming the knife, the bone was reached about two inches above the end of the upper fragment. This was sawed through, and then the operation was completed by pressing through, from above downwards, the large mass of swollen flesh. The great trouble in the operation was in securing the many arteries which gushed forth at almost every cut of the knife, seeming to be much enlarged by the diseased condition. No care was taken to provide a flap, nor was it possible to prepare one. The ligatures were left hanging long, and were brought together in a bundle, while the vast extent of cut surface was contracted as much as possible by very long strips of adhesive plaster drawn over from every direction. Lint and a plaster of simple cerate completed the dressing. The patient was pulseless at the wrist for some time, but reäcted in the course of the evening. I left him next morning tolerably comfortable, after a long sleep induced by opium. Living at a distance of thirty miles, I saw him no more; but he was visited frequently by Dr. Hunter, who afterwards informed me that he recovered rapidly and is now probably living.

Your special question I will answer as well as I can from memory: His age I have stated; his habits I know not, but suppose he was a healthy, well grown boy at the time of the accident. The fracture, I think, was caused by the fall of a tree or branch in the field, and was simple in its character. When the physician was called, if I mistake not, he applied a bandage from the toes to the groin, which remained on till the death of the limb. After the operation, it was carefully examined, and found to be as dry and in as perfect a state of preservation as an Egyptian mummy. The muscles when cut were as hard as wood, and presented the appearance of dried beef. The veins and arteries were entirely obliterated. Altogether, I regarded it as a perfect specimen of dry gangrene, caused by the pressure of the bandage. I have given as faithful a statement of the facts as memory enables me to do, and you may use them according to your judgment.

I am, very respectfully,

Your obedient servant,

R. H. Dalton, M. D.

The following letter, as will be seen, was written subsequently in reply to certain inquiries:

Aberdeen, Mississippi, Oct. 22d, 1856.

Dear Sir: Yours of the 3d instant, has just been received, and I hasten to reply. The time has been so long since the case of Mr. A. B.'s boy occurred, that I may not be exactly correct in some of my statements, as I am altogether dependent on memory for their correctness. But I am certain that he was not more than sixteen or seventeen years old at the time of the amputation. Five or six years after I operated on him, I saw him by accident, and was surprised to find him grown up a large, athletic man, and working at the bootmaker's trade. Another physician being with me, we examined the stump, or rather the scar. Dr. G., who bandaged the limb, was the partner of Dr. H., and I understood that H. only visited the case as G. had left for Virginia. How long the bandage remained on, I do not now recollect, nor am I certain that I heard anything said about a straight splint; the impression, however, on my mind, is that the bandage was not removed for many days after its application, and not till Dr. H. was called in after G.'s departure. The exact position of the fragments, I do not now remember, nor do I suppose that it was possible at that time to have observed it very accurately, for this reason, the integuments were immediately swollen, and no time could be allowed to induce them to subside; hence it would have been difficult to inspect the position of the bone. Having heard the history of the case carefully stated, observing the leg and the lower part of the thigh to be in a state of dry gangrene, and seeing the marks of the bandage visibly impressed on the surface, my opinion was made up at the time (and I so expressed myself) that the gangrene had resulted from pressure of the bandage. The femoral artery at the groin was in a sound and natural state, and, if I mistake not, after the limb was removed, it was traced to the point of obliteration where the gangrene commenced, and where the impression of the bandage was observed; thus far, I think, it was of natural size and calibre. Hence the conclusion is inevitable, that the death of the limb resulted from the pressure of the bandage, and not of one of the fragments. It was a curious specimen of dry mortification, and I regret that I did not use the means of preserving it. I was then engaged in a very laborious practice, thirty miles from home, on horseback, and consequently could not conveniently spare the time to attend to it as an object of surgical curiosity. (Dr. Hunter and myself cut into the leg in various places in order to witness the muscles, arteries, nerves, etc., but found the integuments so hard that it was really difficult to penetrate them with a knife; the resistance to the knife was more like that of dry hickory wood than anything else.) The limb, I suppose, was buried not far from Mr. C.'s house, and the bone, probably, may be found there now, though eighteen years have elapsed since it was removed from the body.

I am yet a practitioner, and take as much interest in the profession, or more, than I ever did, and would be pleased at all times to confer with my brothers on all subjects pertaining to our science, or the art in which we are engaged.

